# Advances in Aerobic Exercise for Pulmonary Arterial Hypertension: Mechanisms of Action and Clinical Insights

**DOI:** 10.3390/jcm15207960

**Published:** 2026-10-15

**Authors:** Tianyu Wang, Yingjie Tan, Limin Deng, Jiang Li

**Affiliations:** 1 Department of Cardiology, The Second Xiangya Hospital of Central South University, Changsha 410011, China; wty092222@163.com (T.W.); 258202069@csu.edu.cn (Y.T.); 2 Department of Nutrition, The Second Xiangya Hospital of Central South University, Changsha 410011, China

**Keywords:** aerobic exercise, pulmonary arterial hypertension, pulmonary vascular remodeling, cardiopulmonary function

## Abstract

Pulmonary arterial hypertension (PAH) is a progressive disease characterized by elevated pulmonary artery pressure and pulmonary vascular resistance, leading to right heart failure and death in severe cases. PAH is classified as Group 1 pulmonary hypertension (PH). Although Groups 2 and 3 PH are more common, this review focuses primarily on PAH. Currently, there are three main classes of targeted drugs in clinical practice, and their main mechanism is to dilate pulmonary artery vessels. These drugs can delay the progression of PAH and improve the quality of life. However, the long-term prognosis is still not optimistic. At present, patients with PAH still need a multi-drug combination and comprehensive treatment. Aerobic exercise, as a non-pharmacological adjunctive therapy, is believed to have a positive impact on PAH treatment when performed regularly in patients with stable conditions. With the widespread application of aerobic exercise in the treatment of cardiovascular diseases, increasing research is exploring its potential in adjunctive therapy for PAH. Studies suggest that exercise-based rehabilitation incorporating aerobic exercise, when added to optimized PAH-targeted therapy, may improve exercise capacity, quality of life, and selected cardiopulmonary or hemodynamic parameters in clinically stable patients with PAH. However, evidence for reversal of pulmonary vascular remodeling or mortality benefit remains limited. This review summarizes the clinical evidence and mechanisms of aerobic exercise specifically in PAH.

## 1. Introduction

Pulmonary hypertension (PH) is a heterogeneous condition classified into five groups. Group 1 PH, termed pulmonary arterial hypertension (PAH), is defined as a mean pulmonary artery pressure (mPAP) > 20 mmHg with pulmonary vascular resistance (PVR) > 2 Wood units and pulmonary artery wedge pressure (PAWP) ≤ 15 mmHg, in the absence of other causes of precapillary PH such as CTEPH or PH associated with lung disease [1]. This review focuses primarily on PAH. When evidence is derived from other PH subtypes, including chronic thromboembolic pulmonary hypertension (CTEPH, Group 4 PH) or mixed PH populations, this is explicitly stated. The pathological changes in PAH mainly affect the distal pulmonary arterioles, characterized by proliferation and inflammation of the pulmonary artery intima, hypertrophic contraction of the media, and fibrosis of the adventitia. Additionally, inflammatory infiltration around pulmonary small blood vessels and distal vascular dilatation or in situ thrombosis led to vascular thickening and plexiform lesions. These pathological changes result in gradually increasing pulmonary artery pressure, which subsequently causes hypertrophy, dilatation, and dysfunction of the right ventricle, ultimately leading to right heart failure and possibly death. Currently, the treatment of PAH mainly focuses on the radical cure of the primary disease and targeted drugs to control the disease. These include three main therapeutic pathways: endothelin receptor antagonists, nitric oxide–soluble guanylate cyclase–cyclic guanosine monophosphate pathway agents, and prostacyclin pathway agents [2]. Targeted drug therapy can effectively dilate blood vessels, delay disease progression, improve prognosis and prolong survival time. However, it does not eliminate the need for non-pharmacological management and rehabilitation. Therefore, adjunctive non-pharmacological strategies, including exercise rehabilitation, are of great interest. However, the precise efficacy of aerobic exercise in PAH still needs further elucidation. This review aims to summarize the clinical evidence and potential mechanisms of aerobic exercise in the treatment of PAH, providing new insights and references for clinical practice.

## 2. Literature Search Methodology

We searched PubMed, Embase, and the Cochrane Library from inception to June 2026 using the following terms: (“pulmonary arterial hypertension” OR “PAH” OR “pulmonary hypertension” OR “PH”) AND (“aerobic exercise” OR “exercise training” OR “rehabilitation” OR “physical training”). Because the review focuses on PAH but the clinical literature frequently combines PAH with CTEPH or other PH subtypes, studies enrolling PAH, CTEPH, or mixed-PH populations were screened when they provided clinically relevant exercise-training data; the PH subtype of each study was recorded and non-PAH evidence was explicitly identified. Only PAH-specific studies were considered direct evidence for PAH; studies involving CTEPH or mixed PH were included only as contextual evidence. Inclusion criteria were: (1) randomized controlled trials, prospective controlled studies, or prospective intervention studies; (2) adult patients with PAH or studies containing a clearly identifiable PAH/CTEPH or mixed-PH population relevant to the review; (3) an exercise-based intervention; and (4) reported clinical outcomes. Exclusion criteria were case reports, reviews, duplicate publications, and studies without relevant clinical endpoints. Studies published in English were considered. Two reviewers independently screened titles and abstracts and assessed potentially eligible full texts, with discrepancies resolved by consensus. EndNote (version 21; Clarivate, Philadelphia, PA, USA) was used for reference management. Thirteen clinical exercise studies were included. This review is narrative rather than a formal systematic review or meta-analysis; therefore, no pooled effect estimates or formal risk-of-bias grading was performed. Instead, study design, population, intervention characteristics, outcomes, and adverse events were critically compared, with particular attention to PAH-specific evidence and the limitations of mixed or non-PAH populations.

## 3. Application of Aerobic Exercise in PAH

Although targeted drugs are widely used in the treatment of PAH, their efficacy has certain limitations. Exercise rehabilitation has attracted increasing attention as a non-pharmacological adjunct in the management of cardiovascular diseases, including PAH. Current recommendations suggest moderate aerobic rehabilitation exercise for patients with stable PAH after achieving stable drug treatment status, which has a positive effect on improving their cardiopulmonary function. In clinical trials involving patients with PAH, aerobic exercise, typically guided by professionals during hospitalization for three weeks at a frequency of 5–7 days/week, is continued at home according to a corresponding regimen after discharge, with a frequency of 3–7 days/week. The intensity of aerobic exercise is usually assessed based on cardiopulmonary exercise testing before treatment, with specific indicators including percentage of peak oxygen consumption (%peak VO_2_), percentage of maximum heart rate (%HRmax), and percentage of heart rate reserve (%HRR). In some prospective studies and randomized controlled trials (RCTs), the intensity classification of aerobic exercise is as Table 1. Although moderate-intensity exercise is commonly used, the available evidence does not establish a single optimal intensity for all patients with PAH; exercise intensity should therefore be individualized according to baseline exercise capacity, right ventricular status, oxygenation, symptoms, and clinical stability [1,3]. Currently the main exercise training used in clinical trials is bicycle ergometer training, and treadmill /ground-based walking, but many studies also include resistance and/or respiratory muscle training [4,5,6,7,8,9,10,11]. Aerobic exercise training during hospitalization should be conducted under the strict supervision of professionals, including monitoring of transcutaneous oxygen saturation, heart rate, and echocardiographic assessment of cardiac function. Several clinical studies of aerobic or aerobic-containing exercise interventions have shown that aerobic exercise training significantly improves six-minute walk distance (6MWD), peak VO_2_, quality of life (QoL), and pulmonary artery systolic pressure (PASP) in patients with PAH [11,12]. The results indicate that moderate aerobic exercise training increases patients’ participation in physical activities, reduces fatigue, and significantly improves their quality of life [5]. Although moderate-intensity exercise is commonly used, there is currently insufficient evidence to determine whether a higher exercise intensity provides greater clinical benefit in patients with PAH. Further randomized studies directly comparing different exercise intensities are needed.

## 4. Clinical Evidence from Exercise Studies in PAH and Other PH Populations

Clinical exercise trials have evaluated patients with PAH, CTEPH, or mixed PH populations. The strongest PAH-relevant evidence comes from randomized controlled studies performed in stable patients receiving disease-targeted therapy, but several studies combine PAH with CTEPH and therefore cannot be interpreted as evidence specific to PAH. Across these studies, exercise training has been associated with improvements in 6MWD, peak VO_2_, physical activity, and quality of life, while selected studies also reported improvements in cardiac output, pulmonary hemodynamics, or right ventricular-related parameters [4,5,6,7,8,9,10,12,13,14,15,16] (Table 2 and Table 3). For example, Fox et al. reported between-group improvements in 6MWD and peak VO_2_ after 12 weeks of ambulatory rehabilitation, whereas Ehlken et al. demonstrated an improvement in peak VO_2_/kg and hemodynamic parameters after 15 weeks of low-dose exercise training [4,10]. The large multicenter trial by Grünig et al. further demonstrated a 34.1 ± 8.3 m between-group improvement in 6MWD, with additional improvements in quality of life, WHO functional class, and peak VO_2_ [11]. These findings support exercise training as an adjunctive strategy for improving functional capacity, but they do not establish mortality benefit or reversal of pulmonary vascular remodeling. Adverse events were reported in two RCTs: Mereles et al. reported dizziness and oxygen desaturation during training [6]; Grünig et al. reported more serious adverse events in the exercise group (e.g., decompensated diabetes, generalized edema), although these were deemed unrelated to exercise [11]. To date, exercise RCTs have primarily focused on two specific PH subtypes, PAH (Group 1) and CTEPH (Group 4), revealing the effectiveness and feasibility of exercise training within these particular subpopulations. In contrast, Groups 2, 3, and 5 PH remain largely unstudied [11,14]. This gap necessitates further research and exploration in the future.

Across the included studies, exercise modalities were heterogeneous. Only a subset evaluated aerobic exercise as a relatively isolated intervention (e.g., treadmill walking, bicycle ergometry, upper-extremity aerobic exercise) [5,7,8,12,14], whereas many protocols combined aerobic exercise with resistance training, respiratory muscle training, stretching, education, or psychosocial components [4,6,9,10,11,15]. Consequently, the observed benefits cannot always be attributed specifically to aerobic exercise. Reported exercise intensity, when specified, ranged from approximately 50% to 80% of peak HR or HRR. Frequency ranged from 2 to 7 sessions per week, and session duration from approximately 10 to 60 min. Intervention periods ranged from 3 to 24 weeks. However, reporting of exercise intensity was inconsistent across the included studies, with only a subset specifying intensity using objective criteria. This heterogeneity limits direct comparison and reproducibility. Future trials should report exercise prescription according to the FITT-VP principle (Frequency, Intensity, Time, Type, Volume, Progression), prespecify progression criteria, and report adherence and adverse events.

The available clinical evidence is limited by small samples, heterogeneous interventions, variable supervision, and frequent use of multicomponent rehabilitation programs. These findings support a beneficial effect on functional capacity but do not establish a disease-modifying effect or mortality benefit. Accordingly, this review gives greater weight to PAH-specific randomized trials and treats CTEPH, mixed-PH, and experimental evidence as supportive or contextual rather than interchangeable with PAH evidence.

## 5. Effects and Mechanisms of Aerobic Exercise in PAH

### 5.1. Improvement of Cardiopulmonary Function Related to PAH by Aerobic Exercise

**Figure 1:**
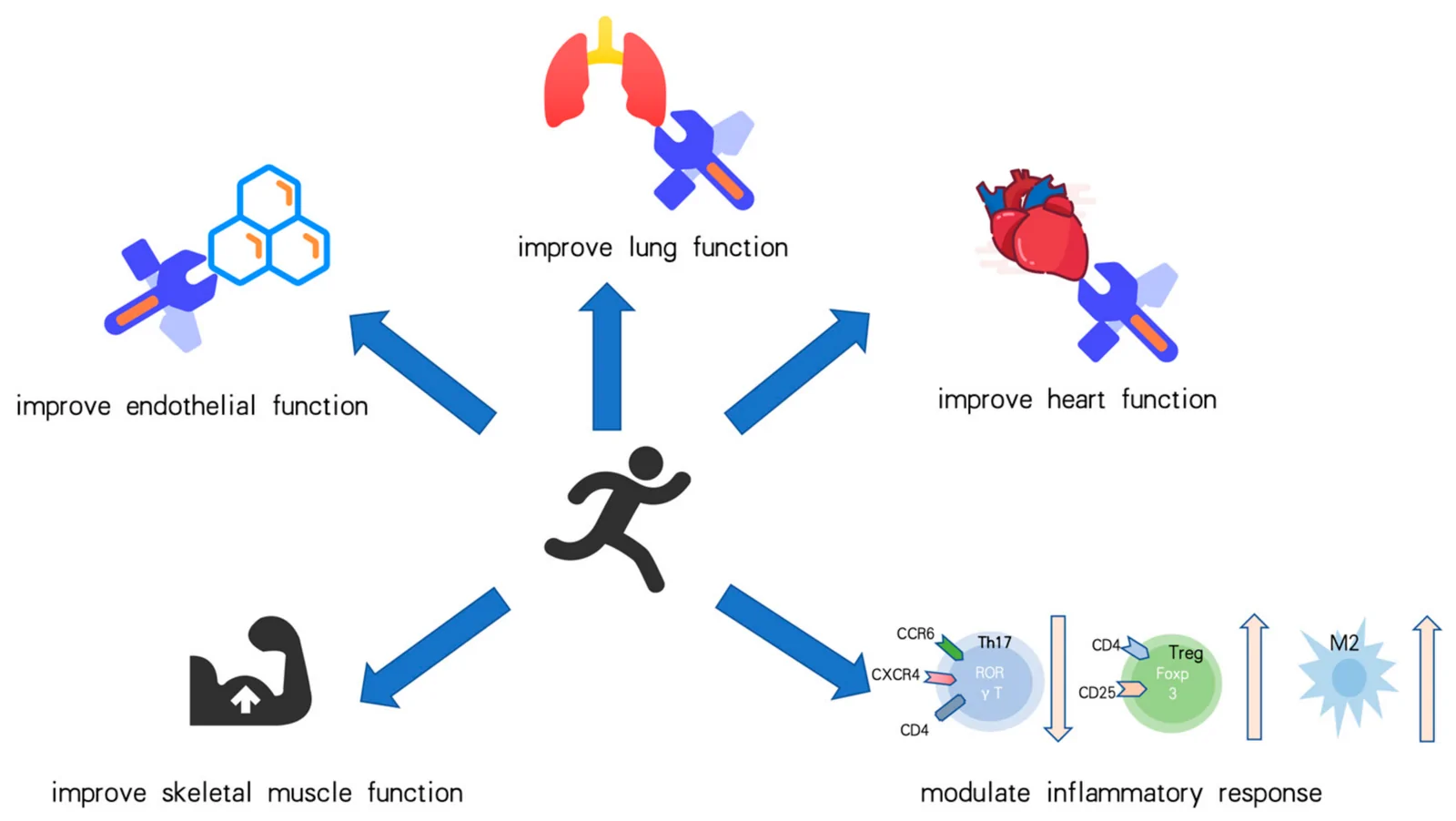
**Potential mechanisms through which aerobic exercise may influence PAH.** Aerobic exercise may improve cardiopulmonary function, skeletal muscle function, endothelial function, and inflammatory signaling. Mechanistic pathways supported mainly by experimental or non-PAH evidence should be interpreted as potential rather than established clinical mechanisms. The arrows indicate the proposed direction of changes in immune cell populations, with a decrease in Th17 cells and increases in Treg cells and M2 macrophages.

Public awareness of aerobic exercise for patients with cardiovascular disease, including PAH, remains insufficient. However, existing research shows that aerobic exercise plays an important role in the management of various cardiovascular diseases. For example, in coronary heart disease, aerobic exercise-based cardiac rehabilitation can significantly reduce cardiovascular mortality, recurrent cardiac events, and hospitalization rates [17]. PAH is a severe cardiac disease with symptoms similar to other cardiovascular diseases, leading to heart failure and even death in severe cases. Most patients with PAH are difficult to cure, and current treatment mainly relies on addressing the underlying disease and using targeted drugs. With the promotion of aerobic exercise rehabilitation in the management of cardiovascular diseases, its application in patients with PAH has gradually increased. Basic research and clinical trials suggest that aerobic exercise may improve functional capacity in PAH [18,19] (Figure 1).

In early PAH, the right ventricle is usually in a compensatory hypertrophic state to adapt to increased pulmonary artery pressure. However, in the late stages of the disease, the right ventricle becomes decompensated, leading to dilatation and increased stress, ultimately exacerbating the disease or causing death due to right heart failure. Factors contributing to right ventricular changes include inflammatory cell infiltration, cytokine release, metabolic changes, and oxidative stress [20].

Aerobic exercise can influence myocardial metabolic function. Myocardial cell energy primarily depends on the oxidative phosphorylation system (OXPHOS) within mitochondria, with fatty acid oxidation (FAO) as the main energy source. During right ventricular hypertrophy, increased systolic wall stress and energy demand, coupled with reduced capillary density and inadequate oxygen supply, necessitate decreased myocardial oxygen consumption and a shift in substrate utilization from FAO to glycolysis. However, in experimental PAH, decreased GLUT4 levels and weakened glycolysis lead to insufficient myocardial energy supply. Additionally, levels of peroxisome proliferator-activated receptor gamma coactivator 1-alpha (PGC-1α), which is involved in mitochondrial biogenesis, and the peroxisome proliferator-activated receptor PPAR-α, and PPAR-γ, which are involved in cardiometabolic regulation, were reduced. In addition, nitration of mitochondrial proteins mainly targets complex V, thereby impairing its activity [18]. Studies have shown that after aerobic exercise, the decrease in GLUT4 levels is attenuated, LDH activity increases, PGC-1α and PPAR-γ levels increase, and complex V nitration is inhibited, thereby improving myocardial metabolism, enhancing cardiac function, and alleviating heart failure symptoms [21,22].

Consistent aerobic exercise may attenuate adverse right ventricular remodeling, right ventricular contractile dysfunction, and calcium cycling disorders caused by PAH. In rats with monocrotaline-induced PAH undergoing aerobic exercise for 3–4 weeks, right heart failure and increased pulmonary artery resistance were delayed, and right ventricular structure and function improved. Rats in the PAH group that underwent aerobic exercise showed decreased hypertrophic index, right ventricular myocardial cell nuclei, type I collagen, and total collagen protein content, as well as decreased transient reactivity of myocardial cells to Ca^2+^ [23]. These findings provide mechanistic support but should not be interpreted as evidence that aerobic exercise reverses pulmonary vascular remodeling in patients with PAH.

Furthermore, aerobic exercise may also improve left ventricular function. PAH leads to increased smooth muscle tension and endothelial cell proliferation in distal pulmonary vessels, subsequently increasing pulmonary vascular resistance and altering right ventricular function and structure. Right ventricular dilatation compresses the interventricular septum, affecting left ventricular compliance and diastolic filling, impairing left ventricular function, and ultimately causing dyspnea and decreased exercise tolerance. Aerobic exercise may improve systemic vascular and left ventricular functional parameters, potentially contributing to improved exercise tolerance in patients with PAH [24].

Aerobic exercise may improve gas exchange function and lung compliance in patients with PAH. In the pathophysiological progression of PAH, dysfunction of the pulmonary vascular endothelium plays an important role, accompanied by significant thickening of the vascular media and adventitial fibrosis, leading to thickening and narrowing of pulmonary arterioles and a marked increase in pulmonary artery resistance. Furthermore, disruptions in pulmonary arteries and bronchi, folding and disorganization of tissue structures, abnormal widening of alveolar septa, extensive inflammatory infiltration, and abnormal deposition of collagen all pose severe challenges to lung function, exacerbating respiratory impairment in patients. In experimental models, regular aerobic exercise has been reported to attenuate pulmonary vascular and airway abnormalities, reduce inflammatory infiltration, and improve structural features related to gas exchange. Additionally, experimental evidence suggests aerobic exercise may reduce vascular wall thickness and lower Type I collagen content, further improve the hemodynamic characteristics of the pulmonary arteries [23]. Moreover, when aerobic exercise is combined with targeted respiratory training, it significantly enhances respiratory muscle strength, improves lung gas exchange capacity, increases peak VO_2_ and 6MWD, improves World Health Organization Functional Class (WHO-FC) of heart function, and elevates quality of life [11,14,25].

### 5.2. Improvement of Skeletal Muscle Function Related to PAH by Aerobic Exercise

Aerobic exercise can inhibit the proteolysis of skeletal muscle in PAH. Research shows that PAH impairs skeletal muscle, leading to muscle atrophy and further affecting patients’ quality of life and exercise capacity [26]. Specifically, levels of atrogin-1 and MuRF1, muscle-specific ubiquitin E3 ligases associated with the ubiquitin-proteasome system (UPS) important in muscle atrophy, are elevated in the quadriceps of patients with PAH. Aerobic exercise reduces atrogin-1 expression, thereby attenuating proteolysis and delaying muscle atrophy progression [27].

Furthermore, aerobic exercise may improve the energy production and utilization of skeletal muscle. Studies have found that PGC-1α increases the expression of mitochondrial aerobic oxidation-related enzymes in skeletal muscle, decreases the expression of glycolytic enzymes, upregulates type 1 fiber-specific proteins, and promotes the conversion of type 2 fibers to type 1 fibers, thereby enhancing skeletal muscle endurance during sustained contraction [28]. Patients with PAH exhibit impaired mitochondrial function and decreased PGC-1α and mitochondrial DNA (mtDNA) expression, resulting in reduced type 1 fiber synthesis. Decreased PGC-1α also reduces microvascular formation and inhibits mitochondrial electron transport chain and oxidative respiration functions, significantly reducing ATP production and affecting normal skeletal muscle function. Animal studies have shown that rats with Sugen/Hypoxia-induced PAH, after aerobic exercise training, exhibited increased mitochondrial supercomplexes in the electron transport chain of their gastrocnemius muscles, improved mitochondrial function, and enhanced ATP production capacity [29] (Figure 1).

### 5.3. Improvement of Inflammation Response Related to PAH by Aerobic Exercise

Aerobic exercise may modulate inflammatory pathways relevant to PAH, including macrophage polarization, the Th17 cell/CD4^+^CD25^+^ regulatory T cell (Treg) balance, and inflammasome signaling. However, the strength of evidence differs substantially across these mechanisms: some observations are derived from human PAH studies, whereas others come from animal or non-PAH disease models. Therefore, these pathways should be regarded as plausible mechanisms rather than established mediators of clinical benefit in PAH. Next, each point is presented separately in detail (Figure 1).

The pathophysiological changes associated with PAH are accompanied by an increase in various inflammatory cytokines, the most common of which are interleukin-1β (IL-1β) and interleukin-6 (IL-6). These inflammatory cytokines directly affect the proliferation, migration, and differentiation of pulmonary artery endothelial cells, smooth muscle cells, and immune cells. Elevated secretion of IL-1β and IL-6 promotes the production of C-C motif chemokine ligand 5 (CCL5), thereby increasing its expression in pulmonary artery endothelial cells, smooth muscle cells, and monocytes. CCL5 exerts chemotactic effects on monocytes and T cells, inducing the recruitment of monocytes at inflammatory sites and promoting the proliferation and dedifferentiation of smooth muscle cells. Additionally, CCL5 can stimulate T cells to secrete endothelin-1, further leading to pulmonary vasoconstriction [20]. In non-PAH models such as breast cancer and obesity, aerobic exercise has been shown to reduce CCL5 and its receptor CCL5R [30,31]. However, whether aerobic exercise has a positive impact on CCL5 levels related to PAH requires further investigation.

Macrophages possess plasticity in response to environmental stimuli and can transform into either pro-inflammatory M1-type or anti-inflammatory M2-type macrophages. M1-type macrophages secrete pro-inflammatory cytokines such as IL-1β, IL-6, and tumor necrosis factor-α (TNF-α), and long-term M1 activation may result in tissue damage. In contrast, M2-type macrophages recruit Tregs and highly express interleukin-10 (IL-10), contributing to inflammation suppression and tissue repair. In physiological states, the conversion between M1 and M2 macrophages is maintained at a certain ratio. However, during PAH, the number of M1-type macrophages increases while the number of M2-type macrophages decreases, thereby promoting inflammation [32,33]. Aerobic exercise helps increase the expression of M2-type macrophages, inhibits inflammatory responses, and exerts a certain inhibitory effect on the progression of experimental PAH models, but direct evidence that aerobic exercise produces a clinically meaningful M1-to-M2 shift in human PAH is currently insufficient [34].

Both Th17 cells and Tregs differentiate from naïve CD4+ T cells, with opposite functions. Th17 cells exert pro-inflammatory effects, while Tregs have anti-inflammatory functions. Furthermore, the pro-inflammatory cytokine interleukin-17 (IL-17) is released by Th17 cells and other immune cells, inducing various inflammatory responses. In physiological conditions, Th17 and Tregs are in balance. However, in the context of PAH, elevated IL-6 and IL-1β lead to reduced FOXP3 (forkhead box P3 transcription factor) expression, resulting in Treg dysfunction and promoting the increase in pro-inflammatory Th17 cells. In non-PAH models such as chronic heart failure, aerobic exercise training has been shown to reduce Th17 levels and increase Treg levels; whether similar effects occur in PAH requires further investigation [35]. Research has found that increased Tregs expression in patients with PAH can promote the expression of cyclooxygenase-2 (COX2), PGI2 synthase (PTGIS), heme oxygenase-1 (HO-1), and programmed death-ligand 1 (PD-L1) in the smooth muscle layer of pulmonary arterioles, thereby increasing PGI2 levels and exerting vasodilatory effects. This provides protection to pulmonary blood vessels and delays disease progression [36].

### 5.4. Improvement of Endothelial Function Related to PAH by Aerobic Exercise

In the early stages of PAH, endothelial cells primarily exhibit a pro-apoptotic state, a pathological process that leads to the loss of the microvascular network. Subsequently, endothelial cells transition to an anti-apoptotic phenotype, a change that can induce plexiform lesions, thereby increasing pulmonary vascular resistance. Ultimately, pulmonary vascular endothelial cells enter a senescent phase, promoting disease progression to an irreversible state [20]. Notably, after undergoing an 8-week aerobic exercise endurance training program, patients with PAH exhibited a significant increase in the levels of circulating progenitor cells (PCs) in their blood. PCs, which originate from the bone marrow, are closely related to endothelial repair function. When the endothelium is damaged, PCs are released into the bloodstream, contributing to the maintenance and restoration of normal endothelial function. Concurrently, aerobic exercise resulted in a decrease in the levels of endothelial cell vesicles (ECVs) in the blood. ECVs, which are vesicles released by endothelial cells in response to damage, inflammation, and oxidative stress, are closely associated with endothelial dysfunction and can exert anti-angiogenic effects by generating reactive oxygen species [37].

The endothelial dysfunction induced by PAH reduces the release and utilization of nitric oxide (NO). Clinical evidence suggests that exercise training may improve markers related to endothelial repair in patients with PAH. In a prospective study of 10 patients with stable PAH, 8 weeks of endurance training increased circulating progenitor cells and reduced endothelial microvesicles, suggesting a shift toward a more favorable endothelial damage–repair balance [37]. Experimental and non-PAH studies have suggested that aerobic exercise may enhance endothelial nitric oxide synthase (eNOS/NO) signaling and modulate endothelial junction proteins such as ZO-1 and ZO-2 [38]. However, whether these mechanisms are directly responsible for endothelial improvement in human PAH remains uncertain.

Additionally, one of the pathological manifestations of PAH is pyroptosis of endothelial cells, which can be caused by NLRP3 (nucleotide-binding domain, leucine-rich-containing family, pyrin domain-containing-3) inflammasome activation in both macrophages and endothelial cells. Under the action of different pathological factors (such as oxidative stress and mitochondrial dysfunction), damage-associated molecular patterns (DAMPs) or pathogen-associated molecular patterns (PAMPs) activate apoptosis-associated speck-like protein (ASC), which in turn activates NLRP3 and induces caspase self-cleavage and activation, leading to the activation of IL-1β and IL-18. NLRP3 also activates the pore-forming protein gasdermin D (GSDMD), resulting in membrane pore formation and the release of inflammatory cytokines extracellularly, participating in pro-inflammatory responses and ultimately causing pyroptosis of endothelial cells [32,39]. Endothelial cells can release and utilize nitric oxide (NO). Studies have shown that NO can inhibit the activation of NLRP3. However, PAH often leads to endothelial dysfunction, resulting in reduced NO release and further increased NLRP3 levels, forming a vicious cycle. Experimental and non-PAH studies suggest that aerobic exercise may attenuate NLRP3 inflammasome activation, with associated reductions in ASC/caspase activation and downstream IL-1β and IL-18 signaling [38,39]. Whether this pathway contributes to the clinical effects of exercise in human PAH remains uncertain (Figure 1).

## 6. Safety Considerations

Exercise in PAH is not risk-free. Potential safety concerns during exercise in PAH include exertional presyncope or syncope, arrhythmias, clinically significant desaturation, chest pain, and worsening right heart failure. In the included clinical trials, however, adverse events were reported in two trials: Mereles et al. reported dizziness and oxygen desaturation during training [6] and Grünig et al. reported more serious adverse events in the exercise group (e.g., decompensated diabetes, generalized edema), although these were deemed unrelated to exercise [11]. Importantly, the absence of frequent serious exercise-related events in clinical trials does not establish that exercise is universally safe, because trial participants were generally selected to be clinically stable and were trained under structured supervision.

Exercise training should generally be initiated in clinically stable patients receiving optimized medical therapy and after individualized risk assessment. Particular caution is required in patients with recent syncope or presyncope, uncontrolled arrhythmias, significant right ventricular failure, or clinically important hypoxemia. During exercise, SpO_2_, heart rate, blood pressure, and symptoms should be monitored, and supplemental oxygen should be considered when clinically indicated (Table 4) [40]. Patients with WHO/NYHA functional class IV disease or severe hemodynamic impairment have been underrepresented in clinical trials, so evidence for safety and efficacy in this population remains uncertain.

## 7. Limitations

This review has several limitations. First, most included clinical studies were small, heterogeneous, and often combined aerobic training with resistance or respiratory training, making it difficult to isolate the effects of aerobic exercise alone. Second, PAH-specific evidence is frequently mixed with CTEPH or other PH populations; therefore, results from non-PAH populations cannot be assumed to apply directly to PAH. Third, many mechanistic insights are derived from animal models or non-PAH diseases, and their translational relevance to human PAH remains uncertain. Fourth, long-term outcomes such as mortality, hospitalization, and sustained disease modification remain insufficiently studied. Fifth, exercise prescription parameters, adherence, progression, and adverse-event reporting were inconsistent across studies. Sixth, although studies were screened by two reviewers, this manuscript is a narrative review and did not perform a formal risk-of-bias assessment or quantitative meta-analysis; publication bias, selection bias, and language bias therefore cannot be excluded. Finally, evidence for PH Groups 2, 3, and 5 is limited.

## 8. Conclusions and Outlook

Current clinical evidence suggests that aerobic or aerobic-containing exercise training may improve exercise capacity, physical activity, and health-related quality of life in clinically stable patients with PAH, particularly when implemented after optimization of targeted therapy and under appropriate supervision. However, the evidence is limited by small sample sizes, heterogeneous and frequently multicomponent interventions, and inconsistent reporting of exercise prescription and adherence. Mechanistic evidence is also heterogeneous and frequently derived from experimental or non-PAH studies.

In clinical practice, exercise rehabilitation should be considered as an adjunct to optimized PAH-targeted therapy rather than a substitute for pharmacological treatment. Appropriate patient selection, initial supervision, monitoring, and individualized exercise prescription are essential. Future studies should determine how exercise modality, intensity, duration, and supervision influence clinical outcomes and whether these effects translate into sustained improvements in hospitalization, disease progression, or survival.

## Figures and Tables

**Table 1:** Reference Exercise Intensity Domains Used in Exercise Prescription.

Intensity	Peak VO_2_ or HRR (%)	HRmax (%)
low intensity	20–39	40–63
moderate intensity	40–59	64–76
high intensity	60–84	76–90

peak VO_2_ = peak oxygen consumption; HRR = heart rate reserve; HRmax = maximum heart rate. These categories are derived from general exercise-prescription literature and should be regarded as reference ranges rather than PAH-specific treatment targets. Individualized intensity selection is preferable in PAH, particularly in patients with right ventricular dysfunction or exercise-induced desaturation. Source: Seo et al., 2021 [3].

**Table 2:** Characteristics of Included Clinical Exercise Studies in PAH and Other PH Populations.

Study	Year	PH Group	Population	Analyzed Sample Size (I/C)	WHO/NYHA	Background PAH Therapy	Intervention Duration (Weeks)	Primary Endpoints/Key Quantitative Findings	Dropout/Adherence
Fox et al. [4]	2011	PAH + CTEPH	PAH (91%) or CTEPH (9%)	11/9	II–III	Yes	12	6MWD: +32 m vs. −26 m (between-group *p* = 0.003). Peak VO_2_: +1.1 vs. −0.5 mL·kg^−1^·min^−1^ (*p* < 0.05).	11/11 vs. 9/11 completed
Weinstein et al. [5]	2013	PAH	PAH	11/13	I–IV	Yes	10	In the training group: 6MWD increased by 53 ± 44 m (*p* = 0.003); treadmill exercise duration increased by 2.1 ± 0.75 min (*p* = 0.001); peak power increased by 25 ± 22 W (*p* = 0.003). Fatigue severity, adjusted activity score, and maximum activity score also improved.	11/14 vs. 13/14 completed
Yılmaz et al. [12]	2020	PAH	PAH	11/11	I–III	Yes	6	Exercise capacity increased ~6.7% and peak VO_2_ ~7% in the training group. Between-group differences: dyspnea *p* < 0.001; peak VO_2_ *p* = 0.031.	NR
Mereles et al. [6]	2006	PAH + CTEPH	PAH (80%) or CTEPH (20%)	15/15	II–III	Yes	15	6MWD +96 ± 61 m vs. −15 ± 54 m; between-group difference 111 m (95% CI 65–139; *p* < 0.001). QoL, WHO functional class, peak VO_2_, VO_2_ at anaerobic threshold, and maximal workload also improved.	NR
Atef et al. [7]	2021	PAH	PAH	14/15	II–III	Yes	12	Reported exercise vs. control values: RVSP 40.21 ± 7.79 vs. 50.29 ± 6.02 mmHg; VO_2_ max 63.65 ± 12.63 vs. 52.66 ± 21.40 mL·kg^−1^·min^−1^; sleep efficiency 89.04 ± 2.75 vs. 78.00 ± 8.76; sleep-quality score 0.40 ± 0.07 vs. 1.67 ± 0.61.	14/15 vs. 15/15 completed
Martínez-Quintana et al. [8]	2010	PAH	PAH associated with CHD	4/4	II–IV	NR	12	No significant improvement was observed in 6MWD, quality of life, handgrip strength, or leg strength.	NR
Ley et al. [16]	2013	PAH + CTEPH	PAH and inoperable CTEPH	10/10	II–III	Yes	3	Reported exercise vs. control values: 6MWD 91 ± 66.2 vs. 16.9 ± 39.8 m; pulmonary flow peak velocity −9.7 ± 8.6 vs. 6.8 ± 15.1 cm/s (*p* = 0.004 and *p* = 0.012, respectively).	10/10 vs. 10/10 analyzed
Ertan et al. [13]	2022	PAH + CTEPH	PAH and CTEPH	12/12	II–III	Yes	8	Endurance capacity, maximal exercise capacity, and weekly step count improved significantly.	12/15 vs. 12/12 completed
Kagioglou et al. [9]	2021	PAH + CTEPH	PAH and CTEPH	12/10	I–III	Yes	24	6MWD +42.8 ± 20.1 m vs. −7.6 ± 10.6 m; between-group 95% CI 36.2–64.6 m. STS-10, STS-20, and lower-limb strength improved.	12/16 vs. 10/16 completed
Babu et al. [14]	2019	Groups 1–5 PH	PH (mixed)	34/33	I–IV	Yes	12	6MWD +48.55 ± 44.98 m with home exercise vs. +13.0 ± 39.8 m in controls (*p* < 0.001). SF-36 physical/mental component scores and WHO functional class improved.	34/42 vs. 33/42 completed
Ehlken et al. [10]	2016	PAH + CTEPH	PAH (*n* = 35), CTEPH (*n* = 11)	38/41	II–IV	Yes	15	Peak VO_2_/kg +3.1 ± 2.7 vs. −0.2 ± 2.3 mL·kg^−1^·min^−1^ (*p* < 0.001), corresponding to ~24.3% vs. 0.9%.	38/46 vs. 41/41 completed
Nagel et al. [15]	2021	PAH + CTEPH	PAH and CTEPH	24/19 with complete hemodynamic data	II–IV	Yes	15	Pulmonary arterial compliance +0.33 ± 0.65 vs. −0.06 ± 1.10 mL/mmHg; difference 0.39 (95% CI 0.15–0.94; *p* = 0.004). Stroke volume +9.9 ± 13.4 vs. −4.2 ± 11.0 mL; difference 14.2 mL (95% CI 6.5–21.8; *p* < 0.001).	NR
Grünig et al. [11]	2021	Groups 1,3,4 PH	PAH (67.2%), CTEPH (12.1%), other (20.7%)	58/58	I–IV	Yes	15	Between-group 6MWD difference 34.1 ± 8.3 m (95% CI 18–51; *p* < 0.0001). SF-36 mental health and peak VO_2_ improved (*p* = 0.004 and *p* = 0.048). WHO class improved in 9 vs. 1 and worsened in 4 vs. 3 (*p* = 0.027).	58/68 vs. 58/61 completed

PAH = pulmonary arterial hypertension; CTEPH = Chronic Thromboembolic Pulmonary Hypertension; I = Intervention; C = Control; 6MWD = six-minute walk distance; peak VO_2_ = peak oxygen consumption; CHD = congenital heart disease; RVSP = right ventricular systolic pressure; QoL = quality of life; VO_2_ max = maximum oxygen consumption; SF-12 = Short Form-12 Item Health Survey; STS = sit-to-stand test. PAH therapy: Yes = PAH-specific therapy reported at baseline; NR = not clearly reported.

**Table 3:** Exercise Prescription Parameters and Safety Characteristics.

Study	Intervention Type	Specific Modalities	Intensity	Frequency (Sessions/Week)	Session Duration (min)	Supervision	Adverse Events
Fox et al. [4]	Aerobic + resistance	Treadmill walking, cycling, step climbing; resistance training	NR	NR	60	Supervised (24 sessions over 12 weeks)	No adverse events occurred during the study.
Weinstein et al. [5]	Aerobic	Treadmill walking	70–80% HRR	3	30–40	Supervised (24–30 sessions over 10 weeks)	NR
Yılmaz et al. [12]	Aerobic	Upper extremity arm ergometer	50–80% of max HR	3	15–45	Supervised	NR
Mereles et al. [6]	Aerobic + resistance + respiratory	Interval bicycle, walking, dumbbell, respiratory training	60–80% of peak HR	5–7	Cycle: 10–25; Walking: 60; Dumbbell: 30; Respiratory: 30	Inpatient (3 weeks) + home-based (12 weeks)	Dizziness in 2; oxygen desaturation in 1
Atef et al. [7]	Aerobic	Bicycle ergometer	60–70% of max HR	3	30–45	Supervised (36 sessions over 12 weeks)	NR
Martínez-Quintana et al. [8]	Aerobic	Bicycle ergometer	NR	2	24	Supervised; 12-week program	No adverse events occurred during the study.
Ley et al. [16]	Aerobic + respiratory	In-hospital respiratory and physical exercise	NR	NR	NR	In-hospital, supervised	NR
Ertan et al. [13]	Aerobic	Ground-based walking	HR ~120 bpm; Borg 3–4	2 supervised + ≥1 unsupervised	30 (increased to 45)	Supervised + unsupervised	NR
Kagioglou et al. [9]	Aerobic + resistance	Cycle/treadmill; resistance exercises; stretching	60–80% of HR at peak VO_2_	3	45–60	Supervised (6 months)	No adverse events occurred during the study.
Babu et al. [14]	Aerobic (home-based)	Home-based exercise	NR	NR	NR	Home-based	No adverse events occurred during the study.
Ehlken et al. [10]	Aerobic + resistance + respiratory	Interval bicycle ergometer, walking, dumbbell, respiratory training	60–80% of HR on CPET (HR < 120 bpm; SpO_2_ > 85%)	Inpatient: 7 (walking/cycling), 5 (resistance/respiratory); Outpatient: 5 (cycling), 2 (walking)	Cycle: 10–25; Walking: 60; Resistance: 30; Respiratory: 30	Inpatient (3 weeks) + home-based (12 weeks)	No adverse events occurred during the study.
Nagel et al. [15]	Aerobic + resistance + respiratory	Cycling, walking, resistance, respiratory training	NR	4–7	NR	Inpatient (3 weeks) + home-based (12 weeks)	NR
Grünig et al. [11]	Aerobic + resistance + respiratory + mental	Cycle, walking, resistance, respiratory, mental training	Cycle 40–60% peak workload	Inpatient: 5–7; Home: 3–7	Cycle: 10–25; Walking: 60; Resistance: 30; Respiratory: 30	Inpatient (10–30 days) + home-based (until 15 weeks)	More serious adverse events in exercise group (e.g., decompensated diabetes, generalized edema), but deemed unrelated to exercise

HRR = heart rate reserve; HR = heart rate; HRmax = maximum heart rate; CPET = cardiopulmonary exercise testing; SpO_2_ = oxygen saturation; NR = not reported. NR does not mean that an event did not occur. SpO_2_ thresholds shown in individual studies are protocol-specific monitoring criteria and should not be interpreted as universal contraindication thresholds.

**Table 4:** Practical Considerations for Exercise Prescription in Stable PAH.

Parameter	Practical Consideration
Patient selection	Clinically stable, optimized PAH therapy
Setting	Initially supervised PAH rehabilitation
Type	Walking/treadmill/cycle ergometry
Frequency	3–5 sessions/week, individualized
Duration	Start low and progressively increase
Intensity	Individualized based on CPET/Borg/HR response
Monitoring	SpO_2_, HR, BP, symptoms
Progression	Gradual increase in duration before intensity
Home training	After initial supervised training and education
Stop/modify	Syncope/presyncope, chest pain, significant desaturation, severe dyspnea, arrhythmia

CPET = cardiopulmonary exercise testing; HR = heart rate; SpO_2_ = oxygen saturation; BP = blood pressure. Suggested framework based on available studies and current guidance; not a universal protocol.

## Data Availability

No new data were created or analyzed in this study. Data sharing is not applicable to this article.

## Abbreviations

PH	pulmonary hypertension
mPAP	mean pulmonary arterial pressure
PAH	pulmonary arterial hypertension
PVR	pulmonary vascular resistance
PAWP	pulmonary artery wedge pressure
CTEPH	chronic thrombo-embolic pulmonary hypertension
OXPHOS	oxidative phosphorylation system
FAO	fatty acid oxidation
Tregs	regulatory T cells
FOXP3	forkhead box P3 transcription factor
IL-10	interleukin-10
TGF-β	transforming growth factor-β
IL-17	interleukin-17
NLRP3	nucleotide-binding domain, leucine-rich-containing family, pyrin domain-containing-3
DAMPs	damage-associated molecular patterns
PAMPs	pathogen-associated molecular patterns
ASC	apoptosis-associated speck-like protein
IL-1β	interleukin-1β
IL-18	interleukin-18
GSDMD	gasdermin D
RCT	randomized controlled trial
6MWD	6-minute walk distance
peak VO_2_	peak oxygen consumption
SF-36	Short Form-36 Item Health Survey
STS	sit-to-stand test
RVSP	right ventricular systolic pressure
QoL	quality of life
PGC-1α	peroxisome proliferator-activated receptor gamma coactivator 1-alpha
TAPSE	tricuspid annular plane systolic excursion
CCL5	C-C motif chemokine ligand 5
PCs	circulating progenitor cells
ECVs	endothelial cell vesicles
NO	nitric oxide
